# The objective structured clinical examination (OSCE) the true origins

**DOI:** 10.1007/s11845-025-04247-1

**Published:** 2026-01-13

**Authors:** Fergus Gleeson

**Affiliations:** Emeritus Associate Professor of Medicine, The Royal College of Surgeons in Ireland (RCSI), Retired Consultant Gastroenterologist, James Connolly Memorial Hospital, Dublin 15, Ireland

**Keywords:** Assessment, Clinical Competence, Dissertation, Objective Structured Clinical Examination (OSCE), Steeplechase

## Abstract

The Objective Structured Clinical Examination (OSCE) first appeared as a title and as an acronym in 1979. It has now become a worldwide phenomenon with almost 3,200 related publications reported in PubMed to date. Prior to its introduction, there were very real concerns regarding Objectivity, Validity and Reliability of prior clinical assessment methods and the overall fairness to students on their assessment of clinical competence. Variations and applications in other allied clinical sub-specialties and non-allied, have now emerged on the method. In addition, numerous psychometric papers have resulted. However, very little in reference to its precise origin has been accurately reported. What has been reported thus far, in some otherwise excellent publications, has been significantly incorrect. A combination of the Steeplechase Examination method, a joint paper from the University of Dundee and the University of Glasgow, and a dissertation for a Diploma in Educational Technology by the author, has resulted in what has now become the globally recognised term: OSCE. The detailed evidence-based narrative of its true origins and its Irish connections to The Royal College of Surgeons in Ireland (RCSI) in this paper summarises the preceding two years of academic endeavour in Dundee prior to its publication debut in January 1979.

Numerous papers have been published about the role, merits and utility of the Objective Structured Clinical Examination (OSCE) but not in any detailed reference to its accurate origin. The nomenclature and acronym entered the world of medical education in 1979 [1]. It might have had its world debut then, but for us, it was the end of the beginning of the story. When we originally drafted the manuscript creating both the title and the associated acronym, OSCE,, it was viewed by us as a contribution to the world of clinical assessment. The fact that the original concept and paper have now assumed a historical position in the field of clinical assessment could not have been predicted and is the understatement of all understatements. Since its original publication, in 1979, a very large volume of subsequent papers has been published. Many, however, have significant errors of fact. The aim of this paper is to establish for perpetuity the true origin of the OSCE and to correct those errors and document the true factual origins of the OSCE and to acknowledge the many contributors involved since its precise publication date of January 1979.

The background process for me personally commenced in 1963 when I commenced my medical studies. At that time, I entered the undergraduate medical faculty of the medical school of the Royal College of Surgeons in Ireland (RCSI). Our course assessments, like many medical schools at that time, took the form of written essays, orals and practical examinations. In the spring of 1965, I had an assessment in histology and rather than the traditional form of practical examination, this was called “The Steeplechase”. The term Steeplechase originated as a horse race over a series of large obstacles dating back to 1752. The race was run in Co. Cork Ireland between two church steeples, hence the name.

While seated at the beginning of the test, the histology steeplechase involved all students moving (up and down through a series of seats) around a fixed series of microscopes with an answer sheet in a laboratory desk setting. Hence, the term “Steeplechase” was used. The origin of the term in medical education is somewhat vague, and currently most frequently used in relation to anatomy and histology assessments [2–4]. The term dates to at least the early 1930s, when it was introduced by Professor William Jessop (Professor of Physiology 1929 -1952) in RCSI [5, 6]. Bernard Towers, who in his own medical school (Liverpool University) had taken the same Steeplechase Exam in the mid 1940’s, subsequently published a paper in the British Medical Journal (BMJ) noting that the term “Steeplechase” was used in many medical schools at that time within the UK [7]. While the term Steeplechase is the most widely used term, in other medical schools, terms such as “spotters”, “bellringers”, and “pin and flag” are alternatives to the term Steeplechase. The term Steeplechase is now mainly used for assessments in Anatomy and Physiology and the OSCE has essentially replaced it in clinical settings. In addition to being used for assessments, it can also be adopted as a teaching/assessment method with the advantage of immediate feedback.

For me, the histology steeplechase methodology, essentially an OSCE by another name, had quite a profound effect on me, such that all the students were assessed on the same histology slides, highlighting the reliability concept in the fact that there was no “luck of the draw” element.

Concerns were increasingly expressed at that time about written, oral and clinical examinations for a variety of reasons. Written examinations were becoming more objective with the introduction of multiple-choice questions. Oral examinations with their lack of objectivity, validity and reliability were increasingly criticised. With respect to the clinical examination, at that time, it was a combination of a long case and a series of short cases. The short cases were essentially included to balance the specialties not covered in the long case. Clinical examinations were largely those of physical examination, mainly to the exclusion of history taking, as identified by Maguire and Rutter [8]. Defects in history taking were clearly identified and the pressure for change was highlighted in the paper by Hampton and colleagues highlighting the relative importance of history taking in diagnosis [9]. The clinical examination was, in general, regarded as the most important component of the assessment. In 1974, a very significant critique of the clinical examination, its defects and recommendations, was very elegantly outlined by John Stokes. Included in his recommendations was the concept of the checklist “to improve reliability” [10]. Postgraduate examinations such as the Membership of the Royal College of Physicians (MRCP) were also undergoing analysis and developments in keeping with the current assessment problems at that time [11, 12]. Differences in marking by examiners led to the establishment of the Hawks/Doves Index and continues in relation to the OSCE [13]. The foregoing is but a limited review of the overall clinical assessment milieu at that time, but of necessity to understand it.

My interest in medical education, in addition to my training in Medicine, continued when I was appointed in my teaching hospital in 1973 as the Tutor/ Registrar in Medicine. During that time, under the supervision of the Professor of Medicine, I was responsible for the organisation and management of undergraduate teaching. In addition, I also obtained very valuable experience organising the MRCP examinations when carried out in my hospital, observing a wide variety of examiners due to the necessity of being close to the action.

During that time, I introduced the Steeplechase methodology for undergraduate clinical teaching and assessment, with the emphasis on immediate feedback to the students. At the end of this two-year period, and with my continuing interest in academic medicine, in 1975, I was appointed as a Lecturer in Medical Education by the University of Dundee, Scotland. At that time, Ronald Harden had moved from Glasgow to Dundee and had set up the Center for Medical Education. I had a 50:50-time breakdown regarding medical education and clinical medicine becoming part of the Senior Registrar Rota. In addition, I had some protected time over a two-year period to obtain a Diploma in Educational Technology, at Dundee College of Educational Technology. In 1975, Ronald Harden and his colleagues in Glasgow, published what he described as a preliminary report on clinical assessment titled “Objective Structured Examination”, subsequently referred to as the “Structured Examination” [14]. With my arrival in Dundee, armed with my own educational experience, in addition to the Steeplechase Exam method, and the preliminary report, the building blocks were in place, for what in time would become the OSCE. Each of the diploma course attendees had to select a major project and the working title for mine was “Assessment of Clinical Competence”. At the conclusion of the two years, for me, it was to become the Objective Structured Clinical Examination. A few weeks later, with the addition of the acronym (see below) it became the OSCE.

Coinciding with my arrival in Dundee, the Center for Medical Education commenced a series of two-week medical education courses/ workshops for senior national and international academics. I was responsible for organising and conducting sessions on the assessment of clinical competence. For these sessions, I included an initial lecture on clinical assessment on what was then referred to in the Centre as the “Structured Exam”. In addition, 20 Station in-course or mock Structured Exams were witnessed by a series of course participants who also depending on the circumstances acted as examiners using pre prepared checklists. Of note, also at this time, simulation was widely established, and the use of simulated/ standarised patients was in some circumstances of great value for exams. Following the mock examinations, the course participants and the undergraduate medical students participated in a discussion on the pros and cons of the exam that had just taken place. The overall discussions were positive but there were concerns about various aspects. The examiners were particularly concerned about the time involved in the preparation and organization of the exam and the production of checklists. In addition, there was significant concern in relation to various issues in their own institutions. For the students, if not briefed about the possibility of moving from a 20-min short cases exam to an 80–100-min exam there was a major concern. However, when the principle of all of the students having an identical exam and highlighting the requirement of detailed briefing for both examiners and students, these concerns were largely allayed.

These recurrent in-course or mock Structured Examinations were also of major benefit to me personally, as over a series of the two-week courses, I was able to highlight the known but more importantly the various potentially unknown problems that could arise in the organisation and production of the Structured Exams. In turn, I was able to document the logistical problems in what would become the first OSCE manuscript that we were writing in parallel with my diploma dissertation [1]. While these issues are now very clear cut, in those days, we were dealing with a very different assessment culture compared to today. For my own diploma and fully recognizing the time and pitfalls involved in the running of a Structured Exam, I initially constructed eleven appendices for dealing with logistics. However, for the 1979 OSCE manuscript itself, we were able by combination to reduce the number to seven.

At that time, the final undergraduate examination at the University of Dundee was in the traditional long and short case format. Professor Alfred Cuschieri had recently arrived as the new Professor of Surgery, in Dundee. While not well versed in this new assessment method, following a discussion with Ronald Harden, he expressed an interest in introducing it. I received a memo from Ronald Harden on the 25th of November 1976 informing me that while Professor Cuschieri was interested, he needed more information about the exam methodology. Ronald requested that I put a plan together for us to further discuss and explain with Professor Cuschieri, the methodology/logistics.

Having completed my own diploma dissertation, I was requested to forward the document to the College of Educational Technology for formal assessment. Up until that time, what was soon to become the OSCE, in the centre, it was still referred to as the Structured Exam. For my own diploma project, I was not satisfied with this nomenclature as I considered it to be vague, and nondescript. For me, it was essential that the term “Objective” would be included in the title, to get away from the subjective “luck of the draw” element. As obvious as it may appear now, I also gave the other words in the title, very serious consideration. In reality, they easily lent themselves to the new title, and so the chosen title for my dissertation was THE OBJECTIVE STRUCTURED CLINICAL EXAMINATION (Fig. 1).

**Fig. 1:**
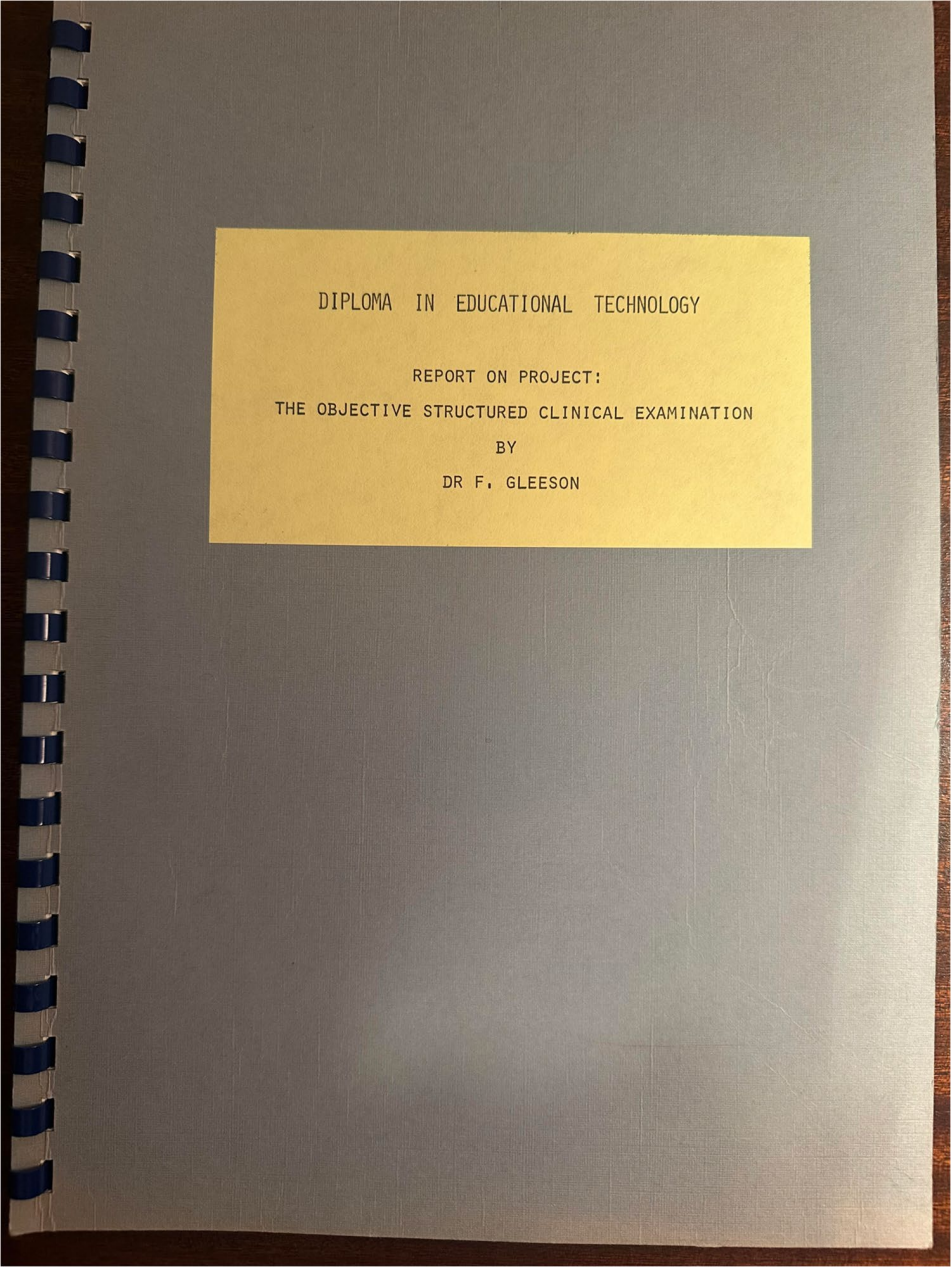
My submitted diploma dissertation title page in 1977 to the Dundee College of Educational Technology entitled “The Objective Structured Clinical Examination”. This was prior to the creation of the OSCE acronym, which I created a few weeks later

Little did I realise at that time that I was creating a dissertation title that would become a frequently used global cross-continent term, and certainly for me, was the understatement of all understatements. However, the project document was quickly returned to me by the College of Educational Technology. The reason for doing so was that the diploma assessors overall were experts in education, in contradistinction to medical education. I was requested therefore to have the project methodology validated by medical experts. To carry out this validation exercise, it was necessary for me to construct a series of questions/ statements which was answered by a group of 12 senior medical academics using a Likert type questionnaire. The series of statements that I constructed all began with the wording The Objective Structured Clinical Examination. Repeating this series, in each of the sentences with the full title, the Objective Structured Clinical Examination was cumbersome and a “real mouthful”, and so the acronym OSCE was born, just as simple as that. Little did I realise at that time that I had been creating a new word in an acronym/jingle that would become a medical educational phenomenon.

Shortly afterwards, I had a telephone conversation with Professor Cuschieri who informed me that the Medical Faculty Board had approved his request to include the new exam format for the upcoming surgery finals and wished to have a further detailed discussion on the precise detailed methodology and the arrangements to deal with the logistics.

I subsequently sent a memo to Ronald Harden (8th February 1977) in reference to the phone call. In his annotated response to my memo, Ronald requested that I put a plan in place to discuss with Professor Cuschieri and while doing so, he would continue to edit what was then the OSCE manuscript that I had just completed and in time published in 1979. We subsequently met with Professor Cuschieri and Mr. Robert Wood, Surgical Senior Lecturer. Robert in turn, together with a few committed surgical senior registrars, and with our plan in place, oversaw the exam production arrangements. The plan was for 20 stations to run concurrently in three separate hospital wards and in “two runs”, during which over 100 final year undergraduate assessments took place in one morning. The examiners were fully briefed, as for many of them, this was a new experience. Similarly, the students were equally briefed. Immediately following the OSCE by which time it was then known, a discussion took place with the examiners, and separately with the students, and there was an overall positive response.

During the afternoon of the exam while the long case part of the surgery exam was taking place, the results of the now completed OSCE were being computed as they were required to be available for the examiner’s meeting that evening. With the necessary administrative personnel in place, the processing of the very large number of examiner checklists and answer sheets of the students took place. The integration and production of the results was handled by Richard Wakeford, who subsequently has had a distinguished career in education at Cambridge University and was very ably assisted by Dr. Robin Cairncross who had joined the Center as a second lecturer a year following me (Fig. 2).

**Fig. 2:**
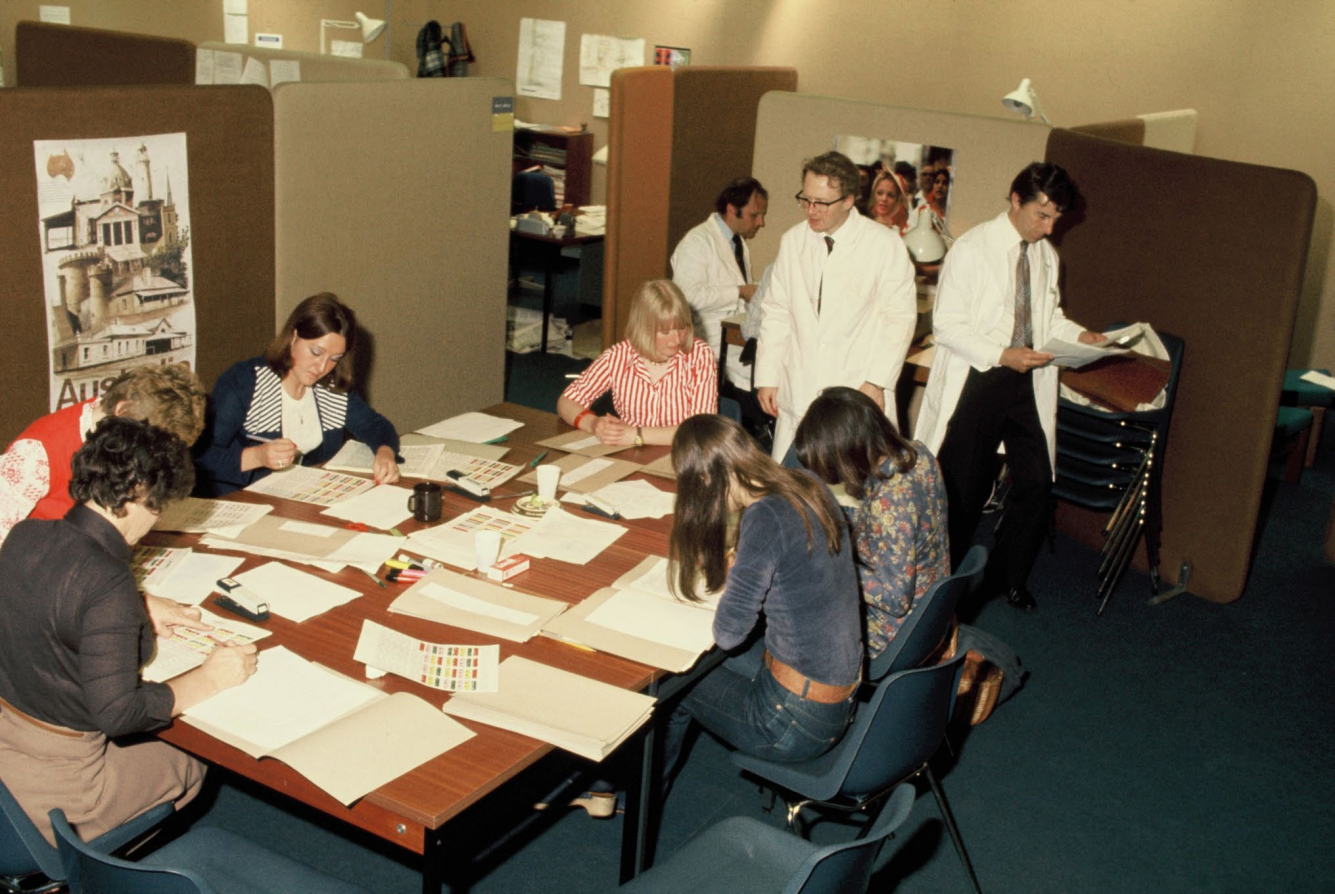
Center for Medical Education Office, Ninewells Hospital, Dundee; The first OSCE in June 1977, with the medical and administrative staff coordinating the OSCE results. From the top left clockwise: 1. Dr. Robin Cairncross, 2. Dr. Fergus Gleeson, 3. Dr. Ronald Harden, 4. Behind Fergus Gleeson was Mr. Richard Wakeford, an Educational Researcher computing the OSCE results

Only those of us intimately involved in the whole process would fully understand and appreciate its initial success but also the reputational risks involved on that Thursday in June 1977. The timing of these developments regarding the OSCE was quite fortuitous. In reference to assessment overall, and particularly to assessment of clinical competence, the unsatisfactory issues as already referred to in the Stokes paper, summarised the situation very well. ^10^ The concepts of objectivity, validity, and reliability were becoming more and more discussed and clear cut. The “luck of the draw” element was clearly not an acceptable phenomenon. Reassurance and transparency for examination candidates in the whole assessment process was necessary.

The newly entitled OSCE itself provided a mechanism to deal with the issues referred to. The timing was just right for such a need and the preliminary report in the paper by Ronald Harden and his colleagues in Glasgow in 1975 was a key development. The timing of my arrival in Dundee and the opportunity to design and execute the dissertation for my diploma was also key. My experience with The Steeplechase, was also hugely important. By allowing the students to trial the in-course or mock OSCE’s allowed for the logistics experience to be accumulated. The positive response of the senior academics attending the two-week courses already referred to was reassuring. The significant acceptance of the OSCE format by Professor Cuschieri and his surgical colleagues cannot be overstated, bearing in mind that at that time the reputational risk for them, should it not have been successful, would have been quite significant [15]. Furthermore, invitations following the series of two-week courses in Dundee to perform medical education workshops including subsequent clinical assessments in Europe, Africa and Asia, were also a key factor. Another key factor was the various academics who came to Dundee for professional sabbaticals. Among these, was the late, highly respected, and widely lamented Ian Hart, Professor of Medicine in Ottawa, Canada. He has rightly been accredited with the introduction of the OSCE in North America. Finally, the personnel in the Center for Medical Education in Dundee were also key, including Mr. William R (Willie) Dunn, Senior lecturer in Education at the University of Glasgow, and visiting consultant advisor to the center. His specific contribution was immense over a broad spectrum of educational topics. Medical schools, all over the world, regularly introduce changes to their curricula for a variety of reasons. Accepting that at least in part, assessment drives the curriculum, the OSCE for its part, has undoubtedly contributed in this respect.

## Epilogue

While it might appear from the foregoing that the OSCE was the answer to all the clinical assessment problems that had existed, that would be incorrect. While significant numbers of examiners at the time of its introduction were overall “early adopters”, others were not so. The move from a more subjective to that of an objective approach was a significant positive for many. Others, however, held the view that their freedom as examiners was being restricted. Some examiners had significant difficulty in adapting to the use of checklists and for some, this amounted to what one examiner described to me as “intellectual boredom”. Another examiner was relieved to be retiring so that he could avoid having to use the OSCE and went so far as to state in a BMJ publication “I am sure that I am not the only examiner who hates it” [16]. The logistical arrangements for some institutions were a significant obstacle to its introduction. Compared to the traditional exam format, a much greater contribution by a variety of personnel, both medical and administrative, was required, in addition to the various economic considerations. More recently, for whatever reason(s), it is regrettable, that one very major organization, the United States Medical Licensing Exam (USMLE) has now, post pandemic permanently discontinued their Multi Station Clinical Skills (Step 2 CS) Sect. (12 clinical encounters each of 15 min) [17]. However, despite these difficulties, the OSCE after almost 50 years has continued to flourish and has stood the test of that time. Assessment is not an island, in addition to the principles of objectivity, validity and reliability, other implications arise, such as practicality. In addition, assessment drives such activities as Learning, Teaching methods and Curriculum planning. Under the concept of the Assessment Utility Index, the OSCE has clearly played its part in this respect [18].

The OSCE was adopted quite rapidly in Ireland as it was elsewhere. My contribution was facilitated by several invitations to speak on the topic and set up OSCE’s in Ireland, the UK and many countries around the world. In 1980 on my appointment as a Consultant Physician I was able to introduce the OSCE in RCSI and as undergraduate and postgraduate Dean in my own hospital James Connolly Memorial Hospital (JCMH), Blanchardstown, Dublin. During the 1980s, with the support of many colleagues, I also set up a Part 2 MRCP (training) course in JCMH attended by many participants from around the world. During these one-week intensive courses I set up two OSCE’s instead of the traditional short cases. I also initiated clinical skills training using the OSCE methodology. In the 1950’s, the pass rate for the MRCP, essentially an exit exam at that time, in the UK was in the region of 15% (https://history.rcp.ac.uk/inspiring-physicians/maurice-henry-pappworth). In the 1980’s, for the MRCPI examination, the average pass rate was in the region of 18%. During that time, I also introduced the Objective Structured Long Examination Record (OSLER) as an alternative to the long case [19]. Such activities had the major benefit of immediate feedback [20]. These intensive dedicated preparatory MRCPI courses resulted in an improved pass rate average of 42% for the course participants who sat for the next MRCPI Part II sitting. It is now also used by a wide variety of allied medical professions such as Nursing, Radiography and Physiotherapy and many other non-medical professions [21]. It has also been incorporated into more modern platforms to include virtual OSCE, powered by artificial intelligence and Chat Generative Pre-trained Transformer (ChatGPT) as new machine learning technological developments evolve, but always bearing in mind the potential pitfalls [22, 23].

For me, the OSCE has essentially replaced the short case problems with a more organised and sophisticated short case multi-station methodology. For others the OSCE, as a single entity, has replaced both the long and short cases in a combination. This for me is to deny the very real validatory importance of the much-criticised long case. In the real everyday world, where clinicians are seeing a new patient for the first time, the obligation and the process of constructing a full workup database is very clearly essential. Without such a database, the construction of a quality product primarily for the patient’s benefit would not exist. That is not to deny that the issues associated with the long case are not very real, but for me, the announcement of its death would be premature. The injury or even death of a patient due to inadequate or poor-quality patient case notes which are essential for a valid long case assessment, is indefensible. I am not alone concerning my belief and support for the long case. The General Medical Council (GMC) recently issued guidance to medical schools in connection with the new UK Medical Licensing Assessment (UKMLA) arrangements. Among their possible options for the Clinical and Professional Skills assessment (CPSA) section is the OSCE, but also the long case option, the Objective Structured Long Examination Record (OSLER) as examples/ suggestions that could be considered for inclusion by the schools [19, 24].

However, while I have now evidence based, documented the detailed genesis and development of the OSCE, by a wide variety of contributors, the origin of the OSLER, is another story, for another day.
